# Essential Oils of Lamiaceae Family Plants as Antifungals

**DOI:** 10.3390/biom10010103

**Published:** 2020-01-07

**Authors:** Tomasz M. Karpiński

**Affiliations:** Department of Medical Microbiology, Poznań University of Medical Sciences, Wieniawskiego 3, 61-712 Poznań, Poland; tkarpin@ump.edu.pl or tkarpin@interia.pl; Tel.: +48-61-854-61-38

**Keywords:** Labiatae, fungi, *Aspergillus*, Cryptococcus, Penicillium, dermatophytes, β-caryophyllene, sesquiterpene, monoterpenes, minimal inhibitory concentration (MIC)

## Abstract

The incidence of fungal infections has been steadily increasing in recent years. Systemic mycoses are characterized by the highest mortality. At the same time, the frequency of infections caused by drug-resistant strains and new pathogens e.g., *Candida auris* increases. An alternative to medicines may be essential oils, which can have a broad antimicrobial spectrum. Rich in the essential oils are plants from the Lamiaceae family. In this review are presented antifungal activities of essential oils from 72 Lamiaceae plants. More than half of these have good activity (minimum inhibitory concentrations (MICs) < 1000 µg/mL) against fungi. The best activity (MICs < 100) have essential oils from some species of the genera *Clinopodium*, *Lavandula*, *Mentha*, *Thymbra*, and *Thymus*. In some cases were observed significant discrepancies between different studies. In the review are also shown the most important compounds of described essential oils. To the chemical components most commonly found as the main ingredients include β-caryophyllene (41 plants), linalool (27 plants), limonene (26), β-pinene (25), 1,8-cineole (22), carvacrol (21), α-pinene (21), p-cymene (20), γ-terpinene (20), and thymol (20).

## 1. Introduction

Fungal infections belong to the most often diseases of humans. It is estimated that about 1.7 billion people (25% of the population) have skin, nail, and hair fungal infections [1]. The development of most of these infections is affected by dermatophytes, namely *Trichophyton* spp., *Microsporum* spp., and *Epidermophyton* spp. [2]. Simultaneously, mucosal infections of the oral and genital tracts caused by *Candida* spp. are very common. About 0.13 billion of women suffer from vulvovaginal candidiasis. On the other hand, oral candidiases are common in babies and denture wearers. Fungi also cause life-threatening systemic infections, with mortality reaching >1.6 million, which is >3-fold more than malaria [3]. Among life-threatening fungal infections prevail cryptococcosis (*Cryptococcus neoformans*) with >1,000,000 cases and mortality rate 20–70%, candidiasis (*Candida albicans*) with >400,000 cases and mortality rate 46–75%, pneumocystosis (*Pneumocystis jirovecii*) with >400,000 cases and mortality rate 20–80%, and aspergillosis (*Aspergillus fumigatus*) with >200,000 cases and mortality rate 30–95% [1,4,5]. In Table 1 are presented diseases caused by some of the most often fungal pathogens among people.

**Figure 1:**
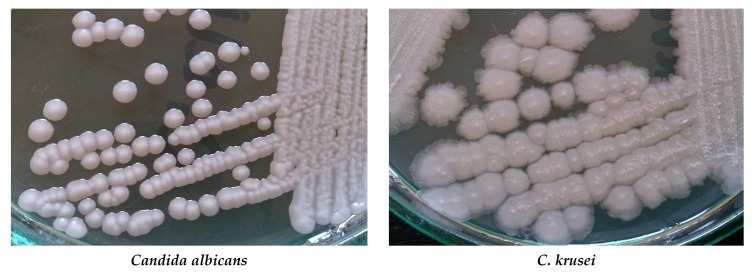

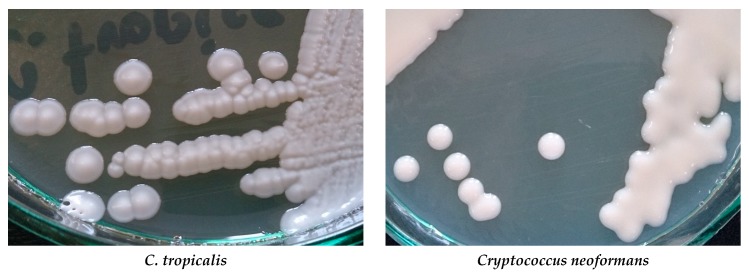
Cultures of selected yeast fungi on Sabouraud agar (Author of photos: Tomasz M. Karpiński).

The big problem is growing drug-resistance amid fungi. Among *Candida* and *Aspergillus* species is observed resistance to azoles, e.g., to fluconazole, voriconazole, and posaconazole. Some *Candida* species, especially *C. glabrata* and *C. parapsilosis,* can be echinocandin- and multidrug-resistant [8,9]. Acquired resistance to echinocandins has also been reported for yeasts *C. albicans*, *C. tropicalis*, *C. krusei*, *C. kefyr*, *C. lusitaniae*, and *C. dubliniensis* [10]. More than 3% of *Aspergillus fumigatus* isolates are resistant to one or more azoles [11]. Polyene resistance mainly concerns amphotericin B. Resistance to this drug is observed in *Fusarium* spp., *Trichosporon* spp., *Aspergillus* spp., and *Sporothrix schenckii* [12,13]. Resistance to amphotericin B has also been reported for *C. albicans*, *C. glabrata*, and *C. tropicalis* [14,15,16]. Cultures of some *Candida* species and *Cryptococcus neoformans* are presented in Figure 1.

The new epidemiological problem is *C. auris*, a multidrug-resistant organism first described in Japan in 2009 [17]. Recently, *C. auris* has been reported from 36 countries from six continents [18]. About 30% of isolates demonstrate reduced susceptibility to amphotericin B, and 5% can be resistant to the echinocandins [19,20]. The estimated mortality from *C. auris* fungemia range from 28% to 60% [21].

Fundamental issues are also the costs of treatment and hospitalization of patients with invasive fungal diseases. According to Drgona et al., all costs range from around €26,000 up to over €80,000 per patient [5].

Therefore, all time, new treatments for fungal infections are being sought. One option may be to apply natural products having antifungal activity. Among these, significant importances have essential oils, which can have a broad antimicrobial spectrum. Rich in the essential oils are among other plants from the Lamiaceae family.

In this review are presented antifungal activities of essential oils from seventy-two (72) plants of the Lamiaceae family. Moreover, are shown the most important compounds of these essential oils. For objective comparison of results, in this paper were included only antifungal studies specifying the minimum inhibitory concentrations (MICs) for essential oils. The MIC (expressed in µg/mL) is the lowest concentration of an antimicrobial agent in which no growth of a microorganism is observed in an agar or broth dilution susceptibility test [22,23,24].

## 2. Components of Essential Oils of Lamiaceae Family

The family Lamiaceae or Labiatae contains many valuable medicinal plants. In the family are 236 genera and between 6900 and 7200 species. To the most abundant genera belong *Salvia* (900 species), *Scutellaria* (360), *Stachys* (300), *Plectranthus* (300), *Hyptis* (280), *Teucrium* (250), *Vitex* (250), *Thymus* (220), and *Nepeta* (200). Lamiaceae plants rich in essential oils have great worth in natural medicine, pharmacology, cosmetology, and aromatherapy [25]. The essential oils are mostly present in leaves, however, they can be found in flowers, buds, fruits, seeds, rind, wood, or roots [26]. Essential oils are mixtures of volatile compounds, which are secondary plant metabolites. They play a role in the defense system of higher plants [27]. Essential oils may contain over 300 different compounds, mainly of molecular weight below 300 [28]. Some oils, e.g., obtained from *Lavandula*, *Geranium*, or *Rosmarinus,* contain 450 to 500 chemicals [29]. Among the active compounds of essential oils are various chemical classes, e.g., alcohols, ethers, aldehydes, ketones, esters, phenols, terpenes (monoterpenes, sesquiterpenes), and coumarins [30,31].

In Table 2 are presented the main chemical components of essential oils of selected Lamiaceae family plants. Plant names were unified according to The Plant List [32], however synonyms used in the literature were also left. Chemical component names were unified, according to PubChem [33].

**Figure 2:**
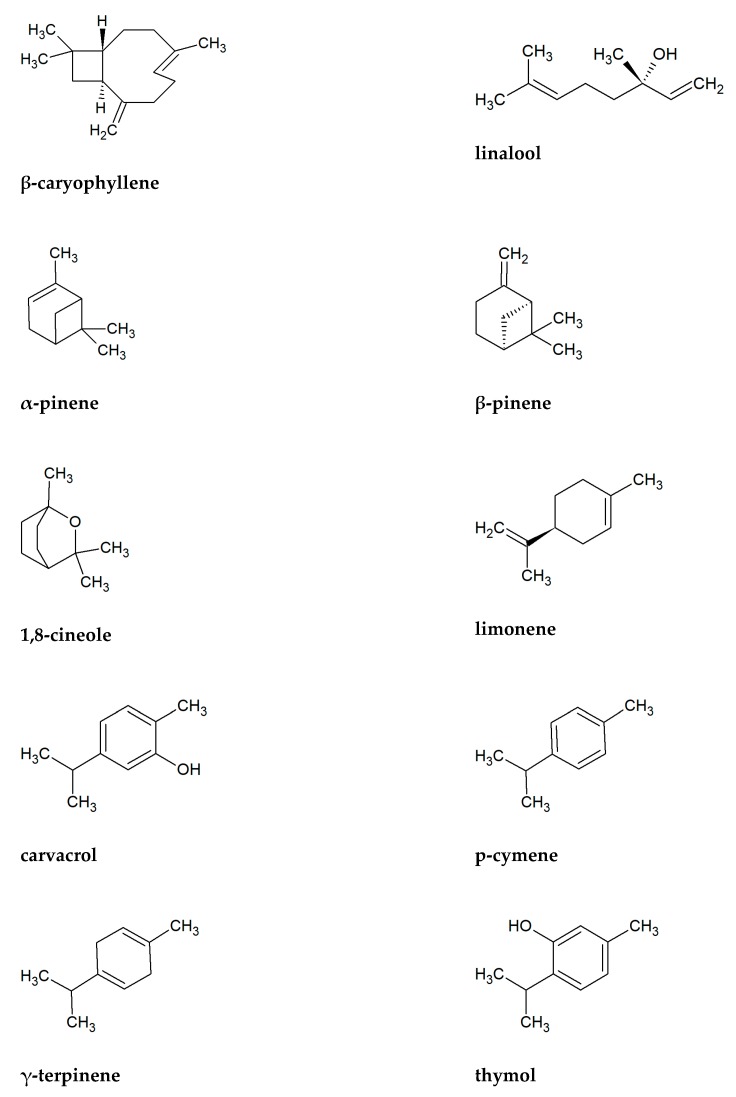
Chemical formulas of ten substances the most commonly found in essential oils of Lamiaceae plants presented in Table 1.

To the chemical components most commonly found as the main ingredients in essential oils, among plants presented in Table 2, include β-caryophyllene (41 plants), linalool (27 plants), limonene (26), β-pinene (25), 1,8-cineole (22), carvacrol (21), α-pinene (21), p-cymene (20), γ-terpinene (20), and thymol (20) (Figure 2). Sesquiterpene β-caryophyllene seems particularly important antifungal component in the Lamiaceae family. Its activity and its derivatives, such as caryophyllene oxide is well known [134,135,136]. According to Bona et al. [137], essential oils containing high concentrations of phenolic monoterpenes (e.g., carvacrol, p-cymene, thymol) have great antifungal activities. Rich in these substances are, among others *Origanum* and *Thymus* plants. Important antifungal chemicals often presented in Lamiaceae are also other monoterpenes as alcohol linalool and cyclic 1,8-cineole, limonene, pinenes, and terpinenes [138,139,140,141,142,143,144,145,146]. Table 1 shows that all of these antifungal substances are common in presented plants.

## 3. Antifungal Activity of Essential Oils of Lamiaceae Family

In Table 3 are shown the antifungal activities of selected Lamiaceae essential oils. More than half of the essential oils have good activity (<1000 µg/mL) against fungi. In some cases are observed significant discrepancies between different studies. An example could be the action of essential oils from Italian *Calamintha nepeta* against *Candida albicans*. In the work of Marongiu et al. [39], minimal inhibitory concentrations amounted to 1.25–2.5 µg/mL, while in Božović et al. [40] MICs were between 780 to 12,480 µg/mL. Differences may be related to the different biochemical composition of the examined essential oils. In results presented by Marongiu et al. [39] the main components of essential oils were pulegone (39.9–64.4%), piperitenone oxide (2.5–19.1%) and piperitenone (6.4–7.7%), while in Božović et al. [40] three main substances were pulegone (37.7–84.7%), crysanthenone (1.3–33.9%) and menthone (0.5–35.4%). Some authors have described that the content of active substances varies depending on the season. In studies of Gonçalves et al. [60] in *Mentha cervina* during the flowering phase in August amount of isomenthone and pulegone in essential oil amounted 8.7% and 75.1% respectively. Simultaneously, in the vegetative phase in February, the content of both components changed significantly and amounted to 77.0% for isomenthone and 12.9% for pulegone. Similarly, Al-Maskri et al. [75] presented essential changes in some compounds of *Ocimum basilicum* essential oil between winter and summer. In the summer essential oil, there is significantly more of linalool, p-allylanisole and β-farnesene, and at the same time much less content of limonene and 1,8-cineole. In this work, a seasonal variation of chemical composition is directly related to other antifungal activities. It is particularly evident in action against *Aspergillus niger*, which was lower in the summer season. Zone of growth inhibition (ZOI) for winter essential oil was 21 mm and MIC > 50 µg/mL, while for summer essential oil-ZOI was 13 mm and MIC > 100 µg/mL [75]. Influence on the content of chemical substances in essential oils also has a method of obtaining them. Ćavar et al. [40] compared the composition of oils obtained from *Calamintha glandulosa* using three methods: Hydrodistillation (HD), steam distillation (SD) and aqueous reflux extraction (ARE). For example, the level of menthone was 3.3% in ARE, 4.7% in HD, and 8.3% in SD method, while for shisofuran was only 0.1% in HD and SD, and even 9.7% in ARE [40]. Additionally, many other factors can affect antimicrobial activity, such as amount and concentration of inoculum, type of culture medium, pH of the medium and incubation time. All these factors can affect the value of MIC [145]. Differences are visible in Table 2. Generally, it can be assumed that the best activity (MICs < 100) have essential oils from *Clinopodium* spp. (excluding *C. nepeta* subsp. *glandulosum* and *C. umbrosum*), *Lavandula* spp., *Mentha* spp. (excluding *M. piperita*), *Thymbra* spp., and *Thymus* spp. (excluding *T. migricus* and *T. vulgaris*). The highest values of MICs are presented among others for *Aeollanthus suaveolens*, *Agastache rugosa*, *Lepechinia mutica*, *Mentha* × *piperita*, and *Salvia sclarea*. Simultaneously, some essential oils have a very different activity, and MIC values differ depending on the region, chemical composition, research methodology, etc. Significant variations can be observed even in *Ocimum basilicum* (MICs 1–10,000), *O. sanctum* (MICs 0.1–500), *Origanum majorana* (MICs 0.5–14,400) or in *Thymus vulgaris* (MICs 0.08–3600).

The mode of action of essential oils is multidirectional. Essential oils lead to disruption of the cell wall and cell membrane through a permeabilization process. The lipophilic compounds of essential oils can pass through the cell wall and damage polysaccharides, fatty acids, and phospholipids, eventually making them permeable [146,147]. Change of the permeability for H^+^ and K^+^ cations affects cellular pH and damage of cellular organelles [148,149]. Additionally, essential oils inhibit the synthesis of fungal DNA, RNA, proteins, and polysaccharides [150]. Essential oils can also disintegrate mitochondrial membrane [151,152]. It has also been shown that essential oil from *Thymus vulgaris* inhibits the production of aflatoxins by *Aspergillus flavus* and leads to the reduction of ergosterol production [123].

## 4. Essential Oils of Lamiaceae Plants in Cosmetics and Medicines

Some essential oils of Lamiaceae family plants and/or their components are commonly used in cosmetics and less often in medicine. Essential oils from *Thymus vulgaris*, *Origanum vulgare*, *Rosmarinus officinalis*, *Calamintha officinalis*, *Salvia officinalis*, or *Lavandula officinalis* are in cosmetic formulations as natural preservatives [187]. *Lavandula angustifolia* oil is commonly used as a fragrance in cosmetics, soaps, perfumes and pharmaceutical products. It also acts as an anti-inflammatory, and is calming, headache relieving, is a sedative and is skin healing. Essential oils from *Lavandula hybrida* and *L. angustifolia* also have anti-louse activity. Compounds (essential oils and mainly menthol) extracted from *Mentha piperita* are commonly used as a fragrance in soaps, cosmetics and as well as in the kitchen as a spice and refreshing products. Moreover, they are often found in chewing gums, toothpastes, and mouthwashes. For medical use, it can be taken orally in gastrointestinal complications. *Rosmarinus officinalis* essential oil is often an ingredient as a fragrance in cosmetics, soaps, bath salts and oils, gels and ointments. It is widely used for hair care and hair-loss treatment because it promotes hair growth and helps against dandruff [188]. In medicine, essential oils from Lamiaceae family are used in aromatherapy (*Salvia sclarea*, *Lavandula officinalis*, *Mentha piperita*, *Rosmarinus officinalis*) [189], sinusitis (*Lavandula officinalis*, *Thymus vulgaris*) [190], and in upper respiratory tract for treatment of catarrh (*Mentha piperita*, *Mentha arvensis*, *Thymus* spp.) [191]. Both essential oils from Lamiaceae plants and mono-substances are used in toothpastes and mouthwashes. In many of these the following chemicals, like limonene, linalool, menthol, and thymol, are presented as flavorings and fragrances [192,193]. Additionally, in some toothpastes are essential oils, e.g., in “Parodontax^®^” occurs *Salvia officinalis* oil, *Mentha piperita* oil, and *Mentha arvensis* oil; in “Lacalut Active Herbal” is *Mentha arvensis* oil, *Thymus vulgaris* oil, and *Salvia officinalis* oil, while in “Signal Family Herbal Fresh” are oils from *Mentha piperita* and *Salvia officinalis* [194]. Literature data confirm a strong antifungal effect against *C. albicans* and anti-inflammatory activity of “Parodontax” toothpaste [195,196]. Besides toothpastes, also some medicines used to rinse the oral cavity or throat contain a large number of essential oils. Mention may be made of “Salviasept” having in its composition the oils from *Mentha × piperita*, *Thymus vulgaris*, *Thymus zygis*, *Origanum majorana,* and *Salvia officinalis* or “Dentosept Complex” containing oils from *Mentha piperita*, *Thymus vulgaris*, *Salvia* sp., *Lavandula* sp., and *Eucalyptus globulus*. Among the antifungal medicines in “Acerin Talk” antifungal foot deodorant are present *Lavandula* sp. oil, menthol, linalool, limonene, and geraniol, while in “Podoflex Tincture” for nails mucosis occur among others oils from *Salvia sclarea* and *Lavandula angustifolia* and mono-substances current in Lamiaceae plants: geraniol, limonene, linalool, citral, and eugenol [194].

## 5. Conclusions

More than half of the essential oils from Lamiaceae family plants have good antifungal activity (MICs < 1000 µg/mL). The microbiological data indicate that they could be used alone or in combination with antifungal drugs in the treatment of fungal infections, especially of the skin and mucous membranes. Some essential oils and their components extracted from Lamiaceae plants are used in cosmetics and medicines. Essential oils may be of future relevance in the treatment of multi-drug resistant fungi.

## Figures and Tables

**Table 1:** Fungal pathogens of humans and most often observed mycoses (based on [6,7]).

**Superficial mycoses**	*Hortae werneckii* (Tinea nigra) *Malassezia furfur* (Pityriasis versicolor) *Piedraia hortae* (Black piedra) *Trichosporon* spp. (White piedra)
**Cutaneous and subcutaneous mycoses**	*Aspergillus* spp. (Onychomycosis, Keratitis) *Candida* spp. (Tinea pedis, Tinea cruris, Onychomycosis, Keratitis) *Chaetomium* spp. (Subcutaneous phaeohyphomycosis) *Curvularia* spp. (Subcutaneous phaeohyphomycosis) *Epidermophyton* spp. (Tinea pedis, Tinea cruris, Onychomycosis) *Exophiala* spp. (Chromoblastomycosis, Subcutaneous phaeohyphomycosis) *Fonsecaea* spp. (Chromoblastomycosis) *Fusarium* spp. (Onychomycosis, Keratitis, Eumycotic mycetoma) *Geotrichum* spp. (Onychomycosis) *Microsporum* spp. (Tinea corporis, Tinea capitis) *Phaeoacremonium* spp. (Eumycotic mycetoma) *Phialophora* spp. (Chromoblastomycosis, Subcutaneous phaeohyphomycosis) *Scopulariopsis brevicaulis* (Onychomycosis) *Sporothrix schenckii* (Lymphocutaneous sporotrichosis) *Trichophyton* spp. (Tinea pedis, Tinea corporis, Tinea cruris, Tinea capitis, Onychomycosis) *Trichosporon* spp. (Onychomycosis)
**Endemic mycoses**	*Blastomyces dermatitidis* (Blastomycosis) *Histoplasma capsulatum* (Histoplasmosis) *Coccidioides immitis/posadasii* (Coccidioidomycosis) *Penicillium marneffei* (Penicilliosis) *Paracoccidioides brasiliensis* (Paracoccidioidomycosis)
**Opportunistic mycoses**	*Acremonium* spp. (Hyalohyphomycosis-cutaneous, disseminated infection) *Alternaria* spp. (Phaeohyphomycosis-subcutaneous, sinusitis, disseminated infection) *Aspergillus* spp. (Allergic reactions, Aspergillosis-nasal, sinusitis, bronchial, pulmonary, systemic dissemination) *Bipolaris* spp. (Phaeohyphomycosis-subcutaneous, sinusitis, brain abscess) *Candida* spp. (Candidiasis-superficial mucosal, cutaneous, widespread hematogenous distribution involving target organs) *Cryptococcus* spp. (Cryptococcosis-cutaneous, pulmonary, meningitis) *Curvularia* spp. (Phaeohyphomycosis-subcutaneous, sinusitis, disseminated infection) *Fusarium* spp. (Hyalohyphomycosis-cutaneous, disseminated infection) *Lichtheimia* spp. (Mucormycosis-cutaneous, invasive) *Mucor* spp. (Mucormycosis-cutaneous, invasive) *Paecilomyces* spp. (Hyalohyphomycosis-cutaneous, disseminated infection) *Pneumocystis jirovecii* (Pneumocystosis-pneumonia, extrapulmonary manifestations) *Rhizomucor* spp. (Mucormycosis-cutaneous, invasive) *Rhizopus* spp. (Mucormycosis-cutaneous, invasive) *Scedosporium* spp. (Hyalohyphomycosis-cutaneous, disseminated infection) *Trichosporon* spp. (Trichosporonosis-invasive disease) *Wangiella* spp. (Phaeohyphomycosis-subcutaneous, sinusitis, brain abscess)

**Table 2:** The main chemical components of the essential oils of selected Lamiaceae family plants.

Essential Oil	Main Chemical Components	References
*Aeollanthus suaveolens* Mart. ex Spreng. = *A. heliotropioides* Oliv.	Linalool (38.5%), α-Farnesene (25.1%), Massoialactone (4.5%), β-Caryophyllene (3.6%), Germacrene D (2.0%)	[34]
*Agastache rugosa* (Fisch. and C.A.Mey.) Kuntze	Methyl chavicol (93.45%), Methyl eugenol (2.48–50.51%), Estragole (8.55%), Eugenol (0.15–7.54%), Thymol (3.62%), Pulegone (2.56%), Limonene (2.49%), β-Caryophyllene (1.19–2.38%),	[35,36]
*Ballota nigra* subsp. *foetida* (Vis.) Hayek	β-Caryophyllene (21.8–22.6%), Caryophyllene oxide (18.0–20.5%), Germacrene D (13.1–16.5%), 2-Hexenal (6.5–11.2%), 1-Octen-3-ol (3.5–5.5%), β-Pinene (1.6–4.4%), Limonene (2.2–4.1%), Linalool (1.2–3.5%), β-Bourbonene (1.5–2.7%), α-Humulene (2.2–2.6%), α-Copaene (1.5–2.2%)	[37]
*Clinopodium dalmaticum* (Benth.) Bräuchler and Heubl = *Micromeria dalmatica* Benth.	Piperitenone oxide (41.77%), Pulegone (15.94%), Piperitenone (10.19%), Limonene (5.77%), Piperitone (3.39%), α-Pinene (2.9%), β-Pinene (2.16%),	[38]
*Clinopodium nepeta* subsp. *glandulosum* (Req.) Govaerts = *Calamintha glandulosa* (Req.) Bentham = *Calamintha officinalis* Moench	Piperitenone (trace–42.6%), Piperitone (0.0–40.3%), Carvone (1–38.7%), Pulegone (0.6–9.7%), Shisofuran (0.1–9.7%), Menthone (trace–8.3%), Dihydrocarveol acetate (0.1–7.6%), Dihydrocarveol (0–6.9%),1,8-Cineole (0.0–6.4%), cis-Carvyl acetate (0.0–6.1%),	[39,40]
*Clinopodium nepeta* (L.) Kuntze = *Calamintha nepeta* (L.) Savi	Pulegone (2.4–84.7%), Isomenthone (1.9–51.3%), Menthone (0.0–35.4%), Crysanthenone (1.3–33.9%), 1,8-Cineole (0.3–21.4%), Piperitenone oxide (0.0–19.1%), Limonene (0.0–13.6%), Isopulegone (0.0–9.4%), Piperitenone (0.0–7.7%), Cinerolone (0.0–5.8%), Isopulegol (0.0–4.1%), Isomenthol (0.0–3.9%), β-Caryophyllene (0.0–3.8%), 3-Octanol (0.0–3.0%), β-Pinene (0.0–2.3%), cis-Piperitone oxide (0.0–2.2%)	[41,42]
*Clinopodium thymifolium* (Scop.) Kuntze = *Micromeria thymifolia* (Scop.) Fritsch	Pulegone (32.81%), Piperitenone (25.7%), Piperitone (11.71%), Isomenthone (4.98%), Limonene (2.4%), β-Caryophyllene (2.39%)	[38]
*Clinopodium umbrosum* (M.Bieb.) Kuntze = *Calamintha umbrosa* Benth.	β-Caryophyllene (13.9%), Germacrene D (11.6%), Spathulenol (10.6%)	[43]
*Dracocephalum heterophyllum* Benth.	Citronellol (74.2%), Geraniol (2.8%), cis-Rose oxide (2.2%), Citronellyl acetate (1.7%)	[44]
*Hymenocrater longiflorus* Benth.	δ-Cadinol (18.49%), α-Pinene (10.16%), p-Menth-1-en-8-ol (9.82%), Hedycaryol (6.42%), β-Eudesmol (4.56%), Spathulenol (4.14%), δ-Cadenene (3.02%), Linalool (2.98%), Caryophyllene oxide (2.81%), β-Bourbonene (2.72%), β-Caryophyllene (2.29%)	[45]
*Hyptis ovalifolia* Benth.	(R)-6-[(Z)-1-Heptenyl]-5,6-dihydro-2H-pyran-2-one (60.0%), γ-Cadinene (6.6%), Viridiflorol (6.08%), Caryophyllene oxide (4.98%), γ-Elemene (4.38%)	[46]
*Hyssopus officinalis* L.	Pinocamphone (5.78–50.77%), 1,8-Cineole (0.47–36.43%), Pinocarvone (0.44–23.4%), β-Pinene (13.38–19.55%), Isopinocamphone (15.32%), α-Phellandrene (trace–3.74%), Sabinene (1.7–2.9%), Myrtenol (1.39–2.7%), α-Pinene (1.01–2.57%), cis-Sabinene hydrate (0.0–2.5%), Myrtenyl methyl ether (1.64–2.1%)	[44,47,48]
*Lavandula angustifolia* Mill.	Linalool (20.18–45.8%), Linalyl acetate (4.6–43.13%), Lavandulyl acetate (0–16.01%), 1,8-Cineole (0.6–13.1%), Camphor (0.52–11.2%), Borneol (0.76–7.5%), Terpinen-4-ol (1.05–5.8%), β-Caryophyllene (0.6–4.95%), Lavandulol (0–3.09%), β-Ocimene (1.5–2.84%), Myrcene (0.4–2.41%)	[49,50,51]
*Lavandula multifida* L.	Carvacrol (41.5–42.8%), β-Ocimene (27.0–27.4%), Myrcene (5.5–5.7%), β-Bisabolene (5.0–5.6%), Terpinolene (2.1–3.1%), α-Farnesene (2.6–2.8%)	[52]
*Lavandula pedunculata* (Mill.) Cav.	Fenchone (6.2–44.5%), 1,8-Cineole (5.1–34.3%), Camphor (8.7–34.0%), β-Pinene (1.4–9.0%), α-Pinene (2.5–8.0%), Camphene (0.8–6.1%), Linalool (0.5–3.8%), Bornyl acetate (0.9–3.5%), Borneol (0.6–3.4%), α-Cadinol (0.2–3.1%), cis-Verbenol (0.2–2.8%), Myrtenal (0.8–2.4%), trans-Verbenol (1.1–2.0%)	[53]
*Lavandula stoechas* L.	Fenchone (0.0–36.2%), 1,8-Cineole (0–33.9%), Camphor (2.2–18%), α-trans-Necrodyl acetate (0.0–17.4%), Lavandulyl acetate (0.0–7.6%), α-trans-Necrodol (0.0–7.1%), Linalool (0.0–6.2%), α-Copaene-8-ol (0.7–4.7%), Viridiflorol (1.4–3.6%), α-Pinene (1.1–3.2%), 2,3,4,4-Tetramethyl-5-methylene-cyclopenten-1-one (0.0–2.8%), Lyratyl acetate (0–2.4%), Myrtenyl acetate (1.0–2.0%), 1,1,2,3-Tetramethyl-4-hidroximethyl-2-cyclopentene (0.0–2.0%)	[51,54]
*Lavandula viridis* L’Her.	1,8-Cineole (34.5–42.2%), Camphor (13.4%), α-Pinene (9.0%), Linalool (6.7–7.9%)	[55]
*Lepechinia mutica* (Benth.) Epling	Δ^3^-Carene (8.69–24.23%), Thujopsan-2-α-ol (0.0–11.9%), Shyobunol (0.0–10.8%), β-Pinene (3.78–7.96%), δ-Cadinene (0.0–6.96%), Globulol (0.0–5.91%), Valerianol (0.0–5.19%), epi-Cubebol (0.0–4.62%), β-Caryophyllene (0.0–4.55%), Limonene (3.79–4.47%), α-Eudesmol (0.0–4.47%), α-Phellandrene (0.34–3.8%), β-Phellandrene (3.79%), γ-Cadinene (0.0–2.86%), α-Pinene (1.23–2.68%), o-Cymene (0.0–2.04%), Isobornyl acetate (0.0–2.2%)	[56,57]
*Marrubium vulgare* L.	γ-Eudesmol (11.93%), β-Citronellol (9.9%), Citronellyl formate (9.5%), Germacrene-D (9.37%), Geranyl formate (6.25%), Geranyl tiglate (5.53%), Ledene (5.35%), 1,8-Cineole (3.72%), Neryl acetate (3.41%), δ-Cadinene (3.3%), Cyclononasiloxane octadecamethyl (3.08%), Geraniol (2.74%), N-trimethylsilyl trifluoroacetamide (2.35%), Eicosamethylcyclodecasiloxane (2.29%), α-Thujone (2.29%), trans-Caryophyllene (2.15%)	[58]
*Melissa officinalis* L.	Geranial (23.4%), Neral (16.5%), Citronellal (13.7%), β-Caryophyllene (4.6%), Geraniol (3.4%), Isomenthone (3.0%), Menthol (2.9%), Methyl citronellate (2.7%), Germacrene D (2.4%), Limonene (2.2%)	[59]
*Mentha cervina* L.	Isomenthone (8.7–77%), Pulegone (12.9–75.1%), Menthone (0.8–4.4%), Limonene (0.8–4.3%)	[60]
*Mentha* × *piperita* L.	Menthol (34.82–43.85%), Menthone (9.1–31.68%), Carvone (0.0–19.54%), Menthyl acetate (1.64–17.4%), Anethole (0.0–9.54%), Isomenthone (4.71–8.08%), Limonene (0.86–6.9%), Menthofuran (6.8%), Eucalyptol (4.36–6.21%), 1,8-Cineole (5.6%), Pulegone (0.47–5.15%), Isomenthol acetate (4.56–4.91%), Isomenthol (0.68–3.58%), Sabinene (0.0–2.5%)	[61,62,63,64]
*Mentha pulegium* L.	Pulegone (2.3–70.66%), Piperitone (0.24–38.0%), Piperitenone (1.58–33.0%), Neomenthol (11.21%), α-Terpineol (0.0–4.7%), 1,8-Cineole (0.11–4.0%), Piperitenone oxide (0.0–3.4%), Menthone (2.63–3.0%), Borneol (0.0–2.9%), Isopulegone (2.33%)	[65,66]
*Mentha requienii* Benth.	Pulegone (77.6%), Isomenthone (18.2%), Limonene (1.76%)	[67]
*Mentha spicata* L.	Pulegone (0.0–78.7%), Carvone (0.0–59.12%), Menthol (0.0–39%), Menthone (5.1–21.9%), Neomenthol (11.2%), Menthyl acetate (0.0–6.9%), Dihydrocarveol (0.0–6.27%), Limonene (1.0–5.8%), 1,8-Cineole (3.0–5.42%), cis-Dihydrocarvone (0.0–4.9%), cis-Carveol (0.0–3.9%), β-Caryophyllene (0.7–2.8%), β-Myrcene (0.3–2.3%)	[49,51,61,68]
*Mentha suaveolens* Ehrh.	Piperitenone oxide (0.0–87.25%), Carvone (0.0–50.59%), Pulegone (0.0–50.0%), Demelverine (0.0–43.46%), Cinerolone (0.0–38.79%), p-Cymenene (0.0–35.22%), Limonene (0.0–31.25%), Piperitone oxide (0.0–26.0%), p-Cymenol-8 (0.0–20.6%), Spathulenol (0.0–18.35%), β-Caryophyllene oxide (0.3–17.25%), α-Pharnesene (0.0–16.54%), α-Cadinol (0.09–10.69%), Calamenene (0.44–10.63%), α-Cubenene (0.0–10.08%), α-Caryophyllene (2.0–9.8%), Veridiflorol (0.0–7.59%), Cubenol (0.0–7.46%), Verbenone (0.0–6.56%), δ-Fenchol (0.3–5.9%), Menthone (0.0–5.7%), Borneol (0.12–5.6%), Citronellyl acetate (0.0–5.45%), δ-Cadinene (0.0–4.89), Eucalyptol (0.0–4.21%), cis-8-Menthene (0.3–4.2%), Fenchone (0.1–3.6%), Geraniol (1.0–3.4%), τ-Muurolol (0.0–3.29%), α-Pinene (0.1–2.7%), β-Caryophyllene (2.56%), cis-Carveol (2.31%), Germacrene D (0.0–2.04%)	[69,70,71]
*Micromeria albanica* (K. Maly) Silic	Piperitenone oxide (38.73%), Pulegone (13.43%), Piperitenone (9.72%), Piperitone (5.62%), Limonene (3.2%), α-Copaene (2.12%)	[38]
*Moluccella spinosa* L.	α-Pinene (26.6%), Caryophyllene oxide (16.8%), β-Caryophyllene (8.6%), α-Thujene (5.9%), Nonacosane (5.5%), Heptacosane (5.3%), Ethylbenzaldehyde (3.4%), Pentacosane (2.5%), Tetracosane (2.3%), Sabinene (2.2%)	[72]
*Nepeta ciliaris* Benth. = *Nepeta leucophylla* Benth.	Caryophyllene oxide (14.8–26.3%), β-Caryophyllene (18.0%), β-Sesquiphellandrene (15.0%), Iridodial b-monoenol acetate (9.8%)	[43]
*Nepeta clarkei* Hook. f.	β-Sesquiphellandrene (22.0%), Actinidine (10.0%), Germacrene D (8.0%)	[43]
*Ocimum basilicum* L.	Linalool (18.0–68.0%), Methyl chavicol (0.0–57.3%), Geraniol (0.0–16.5%), 1,8-Cineole (1.4–15.1%), p-Allylanisole (0.2–13.8%), Eugenol (0.0–12.32%), Limonene (0.2–10.4%), β-Farnesene (0.0–6.3%), τ-Cadinol (trace–5.8%), β-Caryophyllene (0.0–4.5%), α-Bergamotene (0.0–4.34%), α-Cadinol (0.0–4.05%), β-Elemene (0.0–3.62%), δ-Cadinene (0.0–3.6%), Germacrene D (0.0–3.5%), γ-Cadinene (0.0–2.8%), Camphor (0.0–2.4%), β-Myrcene (0.2–2.3%), Terpinen-4-ol (0.0–2.2%), Guaiene (0.0–2.1%), Estragole (0.0–2.03%), Isolimonene (0.0–2.0%), α-Bulnesene (0.0–2.0%), γ-Terpinene (0.0–2.0%)	[64,68,73,74,75,76]
*Ocimum × africanum* Lour. = *Ocimum* × *citriodorum*	Nerol (23.0%), Geranial (15.77%), Methyl chavicol (9.45%), Linalool (9.42%), β-Bisabolenene (8.31%), β-Caryophyllene (7.8%), Geraniol (5.2%), Neral (4.93%), α-Bergamotene (3.52%), α-Bisabolene (2.29%), β-Cubebene (2.26%)	[76]
*Ocimum campechianum* Mill. = *Ocimum micranthum* Willd.	Eugenol (46.55%), β-Caryophyllene (11.94%), β-Elemene (9.06%), 1,8-Cineole (5.35%), δ-Elemene (4.17%), Bicyclogermacrene (2.9%), cis-Ocimene (2.69%), allo-Ocimene (2.42%), α-Humulene (2.4%)	[73]
*Ocimum forskolei* Benth.	endo-Fenchol (31.1%), τ-Cadinol (12.2%), Fenchone (12.2%), Camphor (6.2%), Linalool (5.7%), Methyl(E)-cinnamate (5.1%), α-Bergamotene (3.1%), γ-Cadinene (2.9%), endo-Fenchyl acetate (2.8%), Limonene (2.5%)	[77]
*Ocimum gratissimum* L.	Eugenol (7.42–57.82%), Ethyl cinnamate (0.0–34.0%), Linalool (30.0–32.95%), 1,8-Cineole (6.5–21.91%), α-Bisabolene (0.0–17.19%), Camphor (3.8–11.97%), Thymol (0.0–9.8%), α-Cadinol (5.18%), Germacrene D (0.79–4.76%), α-Terpineol (3.36%), γ-Terpinene (0.0–3.06%), β-Caryophyllene (1.68–3.03%), p-Cymene (0.0–2.11%)	[78,79,80]
*Ocimum tenuiflorum* L. = *Ocimum sanctum* L.	Eugenol (0.0–61.3%), Methyl chavicol (0.0–44.63%), Linalool (0.26–21.84%), α-Caryophyllene (3.3–11.89%), Germacrene D (0.37–9.14%), Carvone (0.0–6.31%), Limonene (0.71–4.39%), β-Caryophyllene (1.4–3.3%), α-Cubebene (0.0–2.54%), Carvacrol (0.0–2.04%)	[81,82,83]
*Origanum compactum* Benth.	Carvacrol (43.26%), Thymol (21.64%), p-Cymene (13.95%), γ-Terpinene (11.28%)	[84]
*Origanum majorana* L.	Terpinen-4-ol (6.66–33.84%), Sabinene hydrate (2.31–28.33%), 1,8-Cineole (0.0–20.9%), Carvacrol (0.0–20.8%), γ-Terpinene (7.59–19.5%), Thymol (0.0–12.18%), α-Terpinene (3.03–10.08%), β-Phellandrene (1.96–8.0%), p-Cymene (2.45–7.84%), Sabinene (3.2–6.7%), Limonene (0.0–5.3%), α-Terpineol (2.7–4.7%), Linalool (0.0–4.4%), Terpinolene (0.98–3.76%), Linalool acetate (1.82–3.2%), Geraniol (2.7%), β-Caryophyllene (1.7–2.38%), α-Pinene (0.0–2.0%)	[62,68,85,86,87]
*Origanum vulgare* L.	Pulegone (0.0–77.45%), Carvacrol (0.21–65.9%), Cymenol (0.0–58.6%), Thymol (3.7–45.22%), o-Cymene (0.0–14.33%), Terpinen-4-ol (0.03–12.55%), β-Terpineol (0.0–10.46%), p-Cymene (0.5–9.3%), γ-Terpinene (3.1–9.12%), Borneol (0.0–6.1%), α-Pinene (0.0–5.1%), Menthone (0.0–4.86%), Linalool (0.0–4.8%), β-Bisabolene (0.0–4.5%), Caryophyllene oxide (0.0–4.5%), Sabinene (0.0–3.91%), β-Phellandrene (0.0–3.74%), β-Caryophyllene (00–3.7%), α-Terpineol (0.0–3.35%), Sabinene hydrate (0.0–3.31%), α-Cadinol (0.0–3.3%), α-Terpinene (1.63–3.1%), Eucalyptol (0.0–2.8%), β-Ocimene (0.0–2.77%), cis-Isopulegone (2.22%), β-Myrcene (0.0–2.2%), Anisole (0.0–2.13%), Piperitenone (0.0–2.13%), Germacrene D (0.0–1.23%)	[49,62,64,68,74,88,89,90,91]
*Pogostemon cablin* (Blanco) Benth.	Patchouli alcohol (38.3–44.52%), α-Bulnesene (0.0–13.3%), δ-Guaiene (12.64%), α-Guaiene (8.89–9.6%), Pogostol (0.0–6.2%), Seychellene (5.8%), α-Bergamotene (5.76%), Eremophilene (4.34%), β-Guaiene (3.54%), β-Caryophyllene (1.93–3.0%), β-Patchoulene (1.8–2.77%)	[92,93]
*Pogostemon heyneanus* Benth.	Acetophenone (51.0%), Patchouli alcohol (14.0%), Nerolidol (5.4%), β-Pinene (5.3%), Limonene (4.0%), Benzoyl acetone (3.1%), α-Pinene (2.4%), β-Caryophyllene (2.0%)	[93]
*Premna microphylla* Turcz.	Blumenol C (49.7%), β-Cedrene (6.1%), Limonene (3.8%), α-Guaiene (3.3%), Cryptone (3.1%), α-Cyperone (2.7%), cis-14-nor-Muurol-5-en-4-one (2.4%)	[94]
*Rosmarinus officinalis* L.	α-Pinene (5.4–37.9%), 1,8-Cineole (0.88–26.54%), Eucalyptol (0.0–24.34%), Limonene (0.0–21.7%), Camphor (2.45–21.6%), Myrcene (0.9–20.18%), Borneol (0.0–18.08%), Bornyl acetete (0.92–14.9%), Verbenone (1.36–12.0%)Camphene (1.7–11.38%), Linalool oxide (0.0–10.8%), β-Pinene (0.0–6.95%), β-Caryophyllene (0.0–6.3%), Linalool (00–5.32%), o-Cymene (0.0–4.43%), p-Cymene (0.0–4.34%), β-Phellandrene (0.0–3.9%), Sabinene (0.0–3.72%), α-Terpineol (1.19–3.36%), Isobornyl acetate (0.0–3.3%), Carvacrol (0.0–3.15%), Verbenol (0.7–3.03%), α-Humulene (0.0–2.6%), α-Terpinene (0.21–2.4%), Terpinen-4-ol (0.34–2.15%)	[51,62,68,87,91,95,96,97,98]
*Salvia fruticosa* Miller	1,8-Cineole (16.9–54.4%), Camphor (0.6–18.34%), Manool (0–11.2%), β-Thujone (0.6–9.0%), β-Pinene (0.0–9.0%), Sabinene (0.0–8.6%), Viridiflorol (0.0–8.4%)β-Caryophyllene (1.53–8.3%), α-Thujone (trace–8.1%), Borneol (0.0–8.0%), Camphene (0.0–7.0%), α-Pinene (1.5–6.85%), Bornyl acetate (0.0–6.8%), α-Terpineol (trace–6.7%), Myrcene (1.3–5.2%), Caryophyllene oxide (0.0–3.9%), α-Terpinyl acetate (0.0–2.2%), α-Humulene (0.16–1.5%)	[49,51,99]
*Salvia mirzayanii* Rech. f. and Esfand	1,8-Cineole (41.2%), Linalool acetate (10.7%), α-Terpinyl acetate (5.7%), Myrcene (4.7%), Geranyl acetate (3.7%), γ-Cadinene (3.3%), Linalool (2.5%), Neryl acetate (2.3%)	[100]
*Salvia officinalis* L.	1,8-Cineole (4.2–50.3%), Camphor (8.8–25.0%), α-Thujone (1.2–19.9%), Viridiflorol (0.5–17.5%), β-Thujone (0.1–9.9%), β-Pinene (0.8–7.3%), β-Caryophyllene (1.4–5.5%), Borneol (1.5–5.4%), α-Pinene (0.5–4.8%), Camphene (0.2–3.9%), Bornyl acetate (0.2–3.3%), α-Terpineol (0.0–3.1%), α-Terpenyl acetate (1.4–2.9%), α-Humulene (0.4–2.6%),α-Farnesene (0.0–2.5%), Eicosane (0.0–2.0%)	[96,101]
*Salvia sclarea* L.	Linalyl acetate (84%), Caryophyllene oxide (24.1%), Linalool (13.6%), 1H-Naphtho(2,1,6)pyran (8.6%), Sclareol (11.5%), Spathulenol (11.4%), β-Caryophyllene (5.1%)	[85,102]
*Satureja hortensis* L.	Thymol (23.12–29.0%), Carvacrol (24.5–26.5%), γ-Terpinene (20.72–22.6%), p-Cymene (6.3–9.3%), α-Terpinene (2.2–2.93%), α-Pinene (2.6–2.91%), β-Pinene (0.92–2.7%), Limonene (0.0–2.55%), β-Bisabolene (0.2–2.2%)	[103,104]
*Satureja montana* L.	Carvacrol (47.1%), p-Cymene (9.0%), γ-Terpinene (6.1%), β-Caryophyllene (3.6%), Linalool (3.1%), Thymol (2.6%), Borneol (2.1%)	[68]
*Satureja thymbra* L.	Thymol (25.16–44.5%), γ-Terpinene (11.1–39.23%), p-Cymene (7.17–21.7%), Carvacrol (4.18–5.3%), Carvacrol methyl ether (0.1–3.33%), α-Terpinene (1.0–3.26%), β-Caryophyllene (1.2–2.76%), Caryophyllene oxide (0.32–2.0%)	[51,105]
*Stachys cretica* L.	Germacrene D (12.9–20.3%), β-Caryophyllene (0.9–9.5%), α-Pinene (0.7–8.6%), Octacosane (0.0–7.2%), β-Pinene (1.5–6.2%), Linalyl acetate (0.0–5.2%), Nonacosane (0.4–4.9%), 9-Geranyl-p-cymene (0.0–4.9%), Heptacosane (0.3–4.8%), cis-Chrysanthenyl acetate (0.0–4.8%), β-Farnesene (3.1–4.0%), Hexadecanoic acid (1.3–3.5%), Caryophyllene oxide (0.5–2.9%), β-Bisabolene (1.6–2.8%), Linalool (0.0–2.6%), Pentacosane (0.0–2.5%), Sesquisabinene (2.1%), Geranyl acetate (0.0–2.1%)	[106]
*Stachys officinalis* (L.) Trevis	Germacrene D (19.9%), β-Caryophyllene (14.1%), α-Humulene (7.5%), δ-Cadinene (4.0%), β-Bourbonene (3.8%), α-Selinene (3.4%), γ-Muurolene (3.2%), Oct-1-en-3-ol (2.9%), Caryophyllene oxide (2.5%), Hexadecanoic acid (2.4%), β-Selinene (2.1%), γ-Cadinene (2.0%), τ-Muurolol (2.0%)	[107]
*Stachys pubescens* Ten.	Germacrene (22.4%), δ-Cadinene (19.7%), 2,6-Octadien (11.5%), Linalool (9.7%), Limonene (6.3%), δ-Elemene (5.4%), β-Ocimene (2.8%), α-Terpinene (2.7%), 2,6-Octadienal (2.1%)	[108]
*Teucrium sauvagei* Le Houerou	β-Eudesmol (28.8%), τ-Cadinol (17.5%), α-Thujene (8.7%), γ-Cadinene (5.6%), Sabinene (4.8%), β-Selinene (4.2%), Limonene (2.8%), γ-Selinene (2.8%), α-Selinene (2.8%), δ-Cadinene (2.2%), Terpinen-4-ol (2.2%), p-Cymene (2.0%),	[109]
*Teucrium yemense* Deflers.	Caryophyllene oxide (4.3–20.1%), 7-epi-α-Selinene (1.3–20.1%), β-Caryophyllene (11.2–19.1%), α-Cadinol (2.0–9.5%), α-Pinene (2.3–6.6%), δ-Cadinene (0.4–6.5%), α-Humulene (4.0–6.4%), τ-Cadinol (2.0–5.7%), γ-Selinene (0.4–5.5%), τ-Muurolol (0.6–4.9%), Shyobunol (0.0–4.6%), Valencene (0.0–3.7%), Ledol (0.5–3.6%), cis-Sesquisabinene hydrate (0.9–3.4%), β-Pinene (1.1–3.1%), Germacrene D-4-ol (0.0–3.1%), γ-Cadinene (0.0–2.7%), β-Selinene (0.3–2.5%), Alloaromadendrene (trace–2.2%)	[77]
*Thymbra capitata* (L.) Cav. = *Thymus capitatus* (L.) Hoffmanns. and Link = *Coridothymus capitatus* (L.) Rchb.f. Solms	Carvacrol (35.6–75.0%), Thymol (0.1–29.3%), p-Cymene (5.0–21.0%), γ-Terpinene (4.0–12.3%), α-Terpinene (1.0–3.0%), β-Myrcene (0.8–3.0%), Linalool (0.5–2.9%), β-Caryophyllene (0.2–2.5%)	[51,110,111,112]
*Thymbra spicata* L.	Carvacrol (20.1–64.0%), γ-Terpinene (11.6–31.2%), p-Cymene (9.6–26.0%), α-Terpinene (1.2–10.1%), β-Myrcene (0.9–7.7%), Thujene (trace–5.2%), β-Caryophyllene (0.5–5.1%)	[51,113,114]
*Thymus bovei* Benth.	Geraniol (35.38%), α-Citral (20.37%), β-Citral (14.76%), Nerol (7.38%), 3-Octanol (4.38%)	[115]
*Thymus daenensis* Celak.	Carvacrol (31.46%), α-Terpineol (22.95%), Thymol (20.2%), Camphene (6.27%), 2,6-Octadien (2.22%), Borneol (2.17%), Cyclohexanone (2.1%)	[108]
*Thymus kotschyanus* Boiss. and Hohen.	Thymol (46.72%), Benzene (6.88%), Carvacrol (3.73%), γ-Terpinene (3.58%), β-Caryophyllene (3.39%), Linalool (2.88%), Phenol (2.61%), Borneol (2.51%), Isopropyl (2.07%)	[108]
*Thymus mastichina* (L.) L.	1,8-Cineole (67.4%), Linalool (4.3%), β-Pinene (4.0%), α-Terpineol (3.5%), α-Pinene (3.0%), Sabinene (2.4%)	[116]
*Thymus migricus* Klokov et Des.-Shost.	Thymol (44.9%), Geraniol (10.8%), γ-Terpinene (10.3%), Citronellol (8.5%), p-Cymene (7.2%)	[117,118]
*Thymus pulegioides* L.	Thymol (26.0%), Carvacrol (21.0%), γ-Terpinene (8.8%), p-Cymene (7.8%), Octan-3-one (3.9%), Camphor (3.9%), β-Bisabolene (3.0%), Borneol (2.9%), Oct-1-en-3-ol (2.0%)	[119]
*Thymus schimperi* Ronniger	Carvacrol (13.91–39.07%), Thymol (11.53–34.66%), o-Cymene (18.72–27.06%), γ-Terpinene (4.13–13.73%), Linalool (3.34–3.59%), 3-Octanone (1.05–2.67%), α-Terpinene (1.67–2.37%)	[120]
*Thymus serpyllum* L.	Thymol (52.6%), p-Cymene (15.3%), β-Caryophyllene (6.8%), Sabinene hydrate (3.8%), γ-Terpinene (2.9%), Terpinen-4-ol (2.4%)	[68]
*Thymus striatus* Vahl.	Thymol (59.5%), γ-Terpinene (11.6%), p-Cymene (6.4%), Carvacrol methyl ether (5.9%), Carvacrol (4.9%), α-Terpinene (3.3%), β-Caryophyllene (2.3%)	[121]
*Thymus vulgaris* L.	Carvacrol (3.5–70.3%), Thymol (0.6–51.8%), Borneol (0.0–40.6%), p-Cymene (2.9–38.9%), o-Cymene (0.0–31.7%), α-Terpineol (0.0–19.9%), Linalool (0.0–16.0%), γ-Terpinene (0.3–12.65%), Camphene (0.0–12.3%), 1,8-Cineole (0.0–11.3%), α-Pinene (0.2–6.1%), β-Caryophyllene (0.0–3.5%), Neomenthol (0.0–2.8%), β-Cubebene (0.0–2.4%), Geraniol (0.0–2.32%), Menthone (0.0–2.2%)	[61,64,74,85,87,104,116,122,123,124,125,126]
*Thymus zygis* L.	Linalool (5.5–39.7%), Thymol (0.52–39.6%), p-Cymene (2.2–21.2%), Terpinen-4-ol (1.0–11.7%), β-Myrcene (3.0–8.6%), γ-Terpinene (7.6–7.9%), α-Terpinene (1.2–4.2%), β-Caryophyllene (1.6–3.6%), α-Pinene (0.9–3.6%), Limonene (1.7–2.6%), Carvacrol (0.08–2.4%), Terpinolene (0.2–2.0%)	[116,127]
*Vitex agnus-castus* L.	Eucalyptol (20.5%), 1,8-Cineole (1.5–19.61%), Bicyclogermacrene (0.0–16.2%), β-Farnesene (0.0–16.1%), Sabinene (0.0–14.57%), Sclarene (0.0–10.9%), α-Pinene (0.9–9.76%), Manool (0.0–8.2%), β-Caryophyllene (3.0–6.6%), β-Caryophyllene oxide (0.0–5.83%), Limonene (0.0–4.89%), Vulgarol B (0.0–4.7%), β-Pinene (0.4–4.4%), α-Terpinyl acetate (1.2–4.21%), β-Sitosterol (3.13%), p-Cymene (0.0–3.11%), Geranyl linalool (0.0–3.1%), β-Phellandrene (0.0–3.0%), Cembrene A (0.7–2.8%), Beyrene (0.0–2.6%), β-Myrcene (trace–2.12%), γ-Elemene (2.11%), s-Cadinol (2.01%)	[51,128,129]
*Zataria multiflora* Boiss.	Thymol (25.8–48.4%), Carvacrol (1.5–34.36%), Carvacrol methyl ether (0.0–28.32%), p-Cymene (2.27–13.2%), γ-Terpinene (0.92–10.6%), Linalool (0.9–6.52%), α-Terpinenyl acetate (5.4%), α-Terpineol (0.5–3.69%), α-Pinene (0.02–3.13%), β-Caryophyllene (2.24–3.12%), Carvacrol acetate (0.0–2.26%), Terpinen-4-ol (0.0–2.21%)	[117,130]
*Ziziphora clinopodioides* L.	Carvacrol (0.63–74.29%), Thymol (7.28–55.6%), γ-Terpinene (1.54–24.56%), p-Cymene (2.21–10.25%), α-Terpinene (0.39–2.77%)	[131,132]
*Ziziphora tenuior* L.	Pulegone (46.8%), p-Menth-3-en-8-ol (12.5%), Isomenthone (6.6%), 8-Hydroxymenthone (6.2%), Isomenthol (4.7%), Limonene (3.2%)	[133]

**Table 3:** Minimal inhibitory concentrations (MICs) of essential oils against fungi.

Source of the Essential Oil	Targeted Fungus	MICs (µg/mL; µl/mL)	Reference(s)
*Aeollanthus suaveolens* Mart. ex Spreng. = *A. heliotropioides* Oliv.	*Candida albicans*	1200–5000	[34]
	*Candida glabrata*	5000	[34]
	*Candida krusei*	2500	[34]
	*Candida parapsilosis*	2500	[34]
	*Candida tropicalis*	1200	[34]
	*Cryptococcus neoformans*	600–5000	[34]
*Agastache rugosa* (Fisch. and C.A.Mey.) Kuntze	*Aspergillus flavus*	10,000	[153]
	*Aspergillus niger*	5000	[153]
	*Blastoschizomyces capitatus*	5000	[153]
	*Candida albicans*	28–5000	[153,154]
	*Candida utilis*	5000	[153]
	*Candida tropicalis*	5000	[153]
	*Cryptococcus neoformans*	10,000	[153]
	*Trichoderma viride*	5000	[153]
	*Trichophyton erinacei*	780	[153]
	*Trichophyton mentagrophytes*	3120	[153]
	*Trichophyton rubrum*	1560	[153]
	*Trichophyton schoenleinii*	1560	[153]
	*Trichophyton soudanense*	1560	[153]
	*Trichophyton tonsurans*	10,000	[153]
	*Trichosporon mucoides*	5000	[153]
*Ballota nigra* subsp. *foetida* (Vis.) Hayek	*Alternaria solani*	750	[37]
	*Botrytis cinerea*	600	[37]
	*Fusarium coeruleum*	350	[37]
	*Fusarium culmorum*	300	[37]
	*Fusarium oxysporum*	300	[37]
	*Fusarium solani*	350	[37]
	*Fusarium sporotrichioides*	350	[37]
	*Fusarium tabacinum*	350	[37]
	*Fusarium verticillioides*	300	[37]
*Clinopodium dalmaticum* (Benth.) Bräuchler and Heubl = *Micromeria dalmatica* Benth.	*Aspergillus niger*	0.4	[38]
	*Aspergillus ochraceus*	0.4	[38]
	*Cladosporium cladosporioides*	0.4	[38]
	*Fusarium tricinctum*	0.4	[38]
	*Penicilium ochrochloron*	0.4	[38]
	*Phomopsis helianthi*	0.2	[38]
	*Trichoderma viride*	0.4	[38]
*Clinopodium nepeta* subsp. *glandulosum* (Req.) Govaerts = *Calamintha glandulosa* (Req.) Bentham = *Calamintha officinalis* Moench	*Aspergillus niger*	1250	[39]
	*Candida albicans*	2500	[39]
*Clinopodium nepeta* (L.) Kuntze = *Calamintha nepeta* (L.) Savi	*Aspergillus flavus*	1.25–10	[41]
	*Aspergillus fumigatus*	0.64–5	[41]
	*Aspergillus niger*	0.32–10	[41]
	*Candida albicans*	1.25–12,480	[41,42]
	*Candida guillermondii*	1.25–2.5	[41]
	*Candida krusei*	1.25–2.5	[41]
	*Candida parapsilosis*	1.25–2.5	[41]
	*Candida tropicalis*	1.25–2.5	[41]
	*Cryptococcus neoformans*	0.32–1.25	[41]
	*Epidermophyton floccosum*	0.64–2.5	[41]
	*Microsporum canis*	0.64–2.5	[41]
	*Microsporum gypseum*	1.25–5	[41]
	*Trichophyton mentagrophytes*	0.64–5	[41]
	*Trichophyton rubrum*	0.64–5	[41]
*Clinopodium thymifolium* (Scop.) Kuntze = *Micromeria thymifolia* (Scop.) Fritsch	*Aspergillus niger*	2	[38]
	*Aspergillus ochraceus*	2	[38]
	*Cladosporium cladosporioides*	2	[38]
	*Fusarium tricinctum*	2	[38]
	*Penicillium ochrochloron*	2	[38]
	*Phomopsis helianthi*	0.4	[38]
	*Trichoderma viride*	2	[38]
*Clinopodium umbrosum* (M.Bieb.) Kuntze = *Calamintha umbrosa* Benth.	*Alternaria solani*	3000	[43]
	*Fusarium oxysporum*	2000	[43]
	*Helminthosporium maydis*	1500	[43]
*Dracocephalum heterophyllum* Benth.	*Alternaria solani*	625	[155]
	*Candida albicans*	625–1000	[44,155]
	*Epidermophyton floccosum*	2500	[155]
	*Fusarium semitectum*	313	[155]
*Hymenocrater longiflorus* Benth.	*Aspergillus niger*	480	[45]
	*Candida albicans*	240	[45]
*Hyptis ovalifolia* Benth.	*Microsporum canis*	15.6–1000	[46,156]
	*Microsporum gypseum*	7.8–1000	[46,156]
	*Trichophyton mentagrophytes*	15.6–1000	[46,156]
	*Trichophyton rubrum*	7.8–1000	[46,156]
*Hyssopus officinalis* L.	*Aspergillus niger*	52,200	[47]
	*Aspergillus ochraceus*	26,100	[47]
	*Aspergillus versicolor*	10,440	[47]
	*Candida albicans*	128–1000	[44,48]
	*Candida glabrata*	512–1024	[48]
	*Candida krusei*	128–256	[48]
	*Candida parapsilosis*	256–512	[48]
	*Candida tropicalis*	512–1024	[48]
	*Cladosporium cladosporioides*	10,440	[47]
	*Cladosporium fulvum*	26,100	[47]
	*Penicillium funiculosum*	52,200	[47]
	*Penicillium ochrochloron*	26,100	[47]
	*Trichoderma viride*	10,440	[47]
*Lavandula angustifolia* Mill.	*Candida albicans*	0.125–512	[50,51,157]
	*Malassezia furfur*	>4	[49]
	*Trichophyton rubrum*	1–512	[49,51]
	*Trichosporon beigelii*	2	[49]
*Lavandula multifida* L.	*Aspergillus flavus*	0.64	[52]
	*Aspergillus fumigatus*	0.32	[52]
	*Aspergillus niger*	0.32	[52]
	*Candida albicans*	0.32	[52]
	*Candida guilliermondii*	0.32	[52]
	*Candida krusei*	0.64	[52]
	*Candida parapsilosis*	0.32	[52]
	*Candida tropicalis*	0.32	[52]
	*Cryptococcus neoformans*	0.16	[52]
	*Epidermophyton floccosum*	0.16	[52]
	*Microsporum canis*	0.16	[52]
	*Microsporum gypseum*	0.16	[52]
	*Trichophyton mentagrophytes*	0.16	[52]
	*Trichophyton mentagrophytes var. interdigitale*	0.16	[52]
	*Trichophyton rubrum*	0.16	[52]
	*Trichophyton verrucosum*	0.16	[52]
*Lavandula pedunculata* (Miller) Cav.	*Aspergillus flavus*	5–10	[53]
	*Aspergillus fumigatus*	2.5–5	[53]
	*Aspergillus niger*	5	[53]
	*Candida albicans*	2.5	[53]
	*Candida guillermondii*	1.25	[53]
	*Candida krusei*	1.25–2.5	[53]
	*Candida parapsilosis*	2.5–5	[53]
	*Candida tropicalis*	1.25–2.5	[53]
	*Cryptococcus neoformans*	0.32–1.25	[53]
	*Epidermophyton floccosum*	0.32–0.64	[53]
	*Microsporum canis*	0.32–1.25	[53]
	*Microsporum gypseum*	0.64–2.5	[53]
	*Trichophyton mentagrophytes*	0.64–1.25	[53]
	*Trichophyton rubrum*	0.32–1.25	[53]
*Lavandula stoechas* L.	*Aspergillus flavus*	1.25–10	[54]
	*Aspergillus fumigatus*	0.64–1.25	[54]
	*Aspergillus niger*	0.32–1.25	[54]
	*Candida albicans*	0.64–512	[51,54]
	*Candida guillermondii*	1.25	[54]
	*Candida krusei*	2.5	[54]
	*Candida parapsilosis*	2.5	[54]
	*Candida tropicalis*	2.5	[54]
	*Cryptococcus neoformans*	0.64	[54]
	*Epidermophyton floccosum*	0.16–0.32	[54]
	*Microsporum canis*	0.16–0.64	[54]
	*Microsporum gypseum*	0.32–0.64	[54]
	*Trichophyton mentagrophytes*	0.32–0.64	[54]
	*Trichophyton mentagrophytes var. interdigitale*	0.16–0.64	[54]
	*Trichophyton rubrum*	0.16–256	[51,54]
	*Trichophyton verrucosum*	0.32	[54]
*Lavandula viridis* L’Her.	*Aspergillus flavus*	5	[55]
	*Aspergillus fumigatus*	2.5	[55]
	*Aspergillus niger*	2.5	[55]
	*Candida albicans*	1.25–2.5	[55]
	*Candida guillermondii*	0.64–1.25	[55]
	*Candida krusei*	1.25–2.5	[55]
	*Candida parapsilosis*	1.25	[55]
	*Candida tropicalis*	1.25–2.5	[55]
	*Cryptococcus neoformans*	0.64	[55]
	*Epidermophyton floccosum*	0.32	[55]
	*Microsporum canis*	0.32	[55]
	*Microsporum gypseum*	0.64	[55]
	*Trichophyton mentagrophytes*	0.32–0.64	[55]
	*Trichophyton mentagrophytes var. interdigitale*	0.32–0.64	[55]
	*Trichophyton rubrum*	0.32	[55]
	*Trichophyton verrucosum*	0.32	[55]
*Lepechinia mutica* (Benth.) Epling	*Candida albicans*	>9000	[56]
	*Fusarium graminearum*	>9000	[56]
	*Microsporum canis*	2200–4500	[56]
	*Pyricularia oryzae*	>9000	[56]
	*Trichophyton rubrum*	2200–4500	[56]
*Marrubium vulgare* L.	*Aspergillus niger*	>1180	[58]
	*Botrytis cinerea*	>1100	[58]
	*Fusarium solani*	>1190	[58]
	*Penicillium digitatum*	>1120	[58]
*Melissa officinalis* L.	*Aspergillus niger*	313	[158]
	*Candida albicans*	30–313	[59,158]
	*Cryptococcus neoformans*	78	[158]
	*Epidermophyton floccosum*	30	[59]
	*Microsporum canis*	30	[59]
	*Penicillium verrucosum*	125	[159]
	*Trichophyton mentagrophytes var. mentagrophytes*	15	[59]
	*Trichophyton rubrum*	15	[59]
	*Trichophyton tonsurans*	15	[59]
*Mentha cervina* L.	*Aspergillus flavus*	2.5–5	[60]
	*Aspergillus fumigatus*	1.25–2.5	[60]
	*Aspergillus niger*	1.25–2.5	[60]
	*Candida albicans*	1.25–2.5	[60]
	*Candida guillermondii*	1.25–2.5	[60]
	*Candida krusei*	1.25–2.5	[60]
	*Candida parapsilosis*	1.25–2.5	[60]
	*Candida tropicalis*	1.25–2.5	[60]
	*Cryptococcus neoformans*	1.25	[60]
	*Epidermophyton floccosum*	0.64–1.25	[60]
	*Microsporum canis*	1.25	[60]
	*Microsporum gypseum*	1.25–2.5	[60]
	*Trichophyton mentagrophytes*	1.25–2.5	[60]
	*Trichophyton rubrum*	1.25	[60]
*Mentha* × *piperita* L.	*Aspergillus flavus*	1450–5000	[62,64]
	*Aspergillus niger*	625–10,000	[64,158]
	*Aspergillus parasiticus*	2500	[64]
	*Candida albicans*	225–1125	[63,158,160]
	*Candida glabrata*	225	[62]
	*Candida tropicalis*	225–230	[62]
	*Cryptococcus neoformans*	313	[158]
	*Fusarium oxysporum*	125	[161]
	*Penicillium chrysogenum*	1250	[64]
	*Penicillium minioluteum*	2050–2200	[62]
	*Penicillium oxalicum*	1300–2050	[62]
	*Penicillium verrucosum*	2500	[90]
*Mentha pulegium* L.	*Aspergillus niger*	0.25–1.25	[65,162]
	*Aspergillus flavus*	1.25	[162]
	*Aspergillus fumigatus*	1.25	[162]
	*Candida albicans*	0.94–3.75	[65,66,162,163]
	*Candida bracarensis*	3.75	[163]
	*Candida guillermondii*	1.25	[162]
	*Candida krusei*	0.94–1.25	[162,163]
	*Candida parapsilosis*	1.25	[162]
	*Candida tropicalis*	1.25	[162]
	*Cryptococcus neoformans*	0.64	[162]
	*Epidermophyton floccosum*	1.25	[162]
	*Microsporum canis*	1.25	[162]
	*Microsporum gypseum*	1.25–2.5	[162]
	*Saccharomyces cervisiae*	<0.3–0.94	[66,163]
	*Trichophyton mentagrophytes*	1.25–2.5	[162]
	*Trichophyton mentagrophytes var. interdigitale*	2.5	[162]
	*Trichophyton rubrum*	1.25	[162]
	*Trichophyton verrucosum*	1.25	[162]
*Mentha requienii* Bentham	*Alternaria* spp.	>40	[67]
	*Aspergillus fumigatus*	>60	[67]
	*Candida albicans*	0.94–40	[67,163]
	*Candida bracarensis*	3.75	[163]
	*Candida krusei*	0.94	[163]
	*Fusarium* spp.	>40	[67]
	*Penicillum* spp.	>60	[67]
	*Rhodotorula* spp.	45	[67]
	*Saccharomyces cerevisiae*	0.94	[163]
*Mentha spicata* L.	*Aspergillus flavus*	1.25	[162]
	*Aspergillus fumigatus*	0.64	[162]
	*Aspergillus niger*	0.64–313	[158,162]
	*Candida albicans*	1.25–625	[51,158,162]
	*Candida guillermondii*	1.25	[162]
	*Candida krusei*	1.25	[162]
	*Candida parapsilosis*	1.25	[162]
	*Candida tropicalis*	1.25	[162]
	*Cryptococcus neoformans*	0.32–313	[158,162]
	*Epidermophyton floccosum*	0.64	[162]
	*Fusarium graminearum*	2.5	[164]
	*Fusarium moniliforme*	2.5	[164]
	*Malassezia furfur*	>4	[49]
	*Microsporum canis*	0.64–2	[68,162]
	*Microsporum gypseum*	0.64–3	[162]
	*Penicillium corylophilum*	0.625	[165]
	*Penicillium expansum*	2.5	[164]
	*Trichophyton erinacei*	3	[68]
	*Trichophyton mentagrophytes*	0.64–3	[68,162]
	*Trichophyton mentagrophytes var. interdigitale*	0.64	[162]
	*Trichophyton rubrum*	0.25–512	[49,51,162]
	*Trichophyton terrestre*	3	[68]
	*Trichophyton verrucosum*	0.32	[162]
	*Trichosporon beigelii*	0.25	[49]
*Mentha suaveolens* Ehrh.	*Candida albicans*	0.34–1250	[69,71,166]
	*Candida glabrata*	0.69–2.77	[69]
	*Cryptococcus neoformans*	300	[167]
	*Microsporum canis*	1250	[167]
	*Microsporum gypseum*	1250	[167]
	*Trichophyton mentagrophytes*	600–1250	[167]
	*Trichophyton rubrum*	5000	[167]
	*Trichophyton violaceum*	600	[167]
*Micromeria albanica* (Griseb. ex K. Maly) Silic	*Aspergillus niger*	0.2	[38]
	*Aspergillus ochraceus*	0.2	[38]
	*Cladosporium cladosporioides*	0.2	[38]
	*Fusarium tricinctum*	0.4	[38]
	*Penicilium ochrochloron*	0.2	[38]
	*Phomopsis helianthi*	0.2	[38]
	*Trichoderma viride*	0.4	[38]
*Moluccella spinosa* L.	*Aspergillus niger*	50	[72]
	*Candida albicans*	100	[72]
	*Fusarium oxysporum*	100	[72]
*Nepeta ciliaris* Benth. = *Nepeta leucophylla* Benth.	*Alternaria solani*	3000	[43]
	*Candida albicans*	0.78	[168]
	*Fusarium oxysporum*	1000	[43]
	*Trichophyton rubrum*	0.19	[168]
	*Helminthosporium maydis*	1500	[43]
*Nepeta clarkei* Hook. f.	*Alternaria solani*	3000	[43]
	*Fusarium oxysporum*	2000	[43]
	*Helminthosporium maydis*	2000	[43]
*Ocimum basilicum* L.	*Aspergillus flavus*	10,000	[64]
	*Aspergillus fumigatus*	>50	[75]
	*Aspergillus niger*	>50–10,000	[64,75,158]
	*Aspergillus parasiticus*	5000	[64]
	*Candida albicans*	30–625	[73,74,158]
	*Candida guilliermondii*	3.125–6.25	[76]
	*Cryptococcus neoformans*	313–1250	[158,169]
	*Debaryomyces hansenii*	6.25	[76]
	*Epidermophyton floccosum*	15	[74]
	*Microsporum canis*	1–15.2	[68,74]
	*Microsporum gypseum*	3	[68]
	*Penicillium chrysogenum*	10,000	[64]
	*Penicillium italicum*	>50	[75]
	*Rhizopus stolonifer*	>50	[75]
	*Rhodotorula glutinis*	86	[73]
	*Trichophyton erinacei*	2.5	[68]
	*Trichophyton mentagrophytes*	2.5–8.3	[68,74]
	*Trichophyton terrestre*	3	[68]
	*Saccharomyces cerevisiae*	28	[73]
	*Schizosaccharomyces pombe*	86	[73]
	*Trichophyton rubrum*	8.3	[74]
	*Trichophyton tonsurans*	8	[74]
	*Yarrowia lypolytica*	57	[73]
*Ocimum × africanum* Lour. = *Ocimum* × *citriodorum*	*Candida guilliermondii*	3.125	[76]
	*Debaryomyces hansenii*	1.56	[76]
*Ocimum campechianum* Mill. = *Ocimum micranthum* Willd.	*Candida albicans*	69	[73]
	*Rhodotorula glutinis*	139	[73]
	*Saccharomyces cerevisiae*	69	[73]
	*Schizosaccharomyces pombe*	104	[73]
	*Yarrowia lypolytica*	69	[73]
*Ocimum forskolei* Benth.	*Candida albicans*	35.3–8600	[77,170]
*Ocimum gratissimum* L.	*Aspergillus fumigatus*	>1000	[78]
	*Candida albicans*	350–1500	[78,171]
	*Candida krusei*	750	[171]
	*Candida parapsilosis*	380	[171]
	*Candida tropicalis*	1500	[171]
	*Cryptococcus neoformans*	250–300	[78,79]
	*Fusarium oxysporum f.* sp. *cubense*	62.5	[80]
	*Fusarium oxysporum f.* sp. *lycopersici*	31.25	[80]
	*Fusarium oxysporum f.* sp. *tracheiphilum*	62.5	[80]
	*Fusarium solani*	62.5	[80]
	*Macrophomina phaseolina*	62.5–125	[80]
	*Malassezia pachydermatis*	300	[78]
	*Microsporum canis*	200–500	[78,172]
	*Microsporum gypseum*	150–250	[78,172]
	*Rhizoctonia solani*	31.25	[80]
	*Scopulariopsis brevicaulis*	400	[78]
	*Trichophyton interdigitale*	250	[78]
	*Trichophyton mentagrophytes*	200–250	[78,172]
	*Trichophyton rubrum*	150–250	[78,172]
*Ocimum tenuiflorum* L. = *Ocimum sanctum* L.	*Aspergillus flavus*	300	[83]
	*Candida albicans*	0.1–300	[81,82]
	*Candida glabrata*	0.15–300	[81,82]
	*Candida krusei*	0.35–450	[81,82]
	*Candida parapsilosis*	0.25–500	[81,82]
	*Candida tropicalis*	0.1–300	[81,82]
*Origanum compactum* Benth.	*Alternaria alternata*	300	[84]
	*Bipolaris oryzae*	300	[84]
	*Fusarium equiseti*	300	[84]
	*Fusarium graminearum*	300	[84]
	*Fusarium verticillioides*	300	[84]
*Origanum majorana* L.	*Aspergillus flavus*	450–650	[62]
	*Aspergillus niger*	625	[158]
	*Botrytis cinerea*	5000	[87]
	*Candida albicans*	625	[158]
	*Cryptococcus neoformans*	313	[158]
	*Fusarium delphinoides*	1800–14,400	[85]
	*Fusarium incarnatum-equiseti*	450–3600	[85]
	*Fusarium napiforme*	3600–14,400	[85]
	*Fusarium oxysporum*	900–3600	[85]
	*Fusarium solani*	900–3600	[85]
	*Fusarium verticillioides*	14,400	[85]
	*Microsporum canis*	0.5	[68]
	*Microsporum gypseum*	2	[68]
	*Penicillium expansum*	10,000	[87]
	*Penicillium minioluteum*	400–500	[62]
	*Penicillium oxalicum*	350–400	[62]
	*Sporothrix brasiliensis*	≤2250–9000	[86]
	*Sporothrix schenckii*	≤2250–9000	[86]
	*Trichophyton erinacei*	1	[68]
	*Trichophyton mentagrophytes*	1.5	[68]
	*Trichophyton terrestre*	2	[68]
*Origanum vulgare* L.	*Aspergillus flavus*	0.64–2500	[64,89,91]
	*Aspergillus fumigatus*	0.32–0.64	[89]
	*Aspergillus niger*	0.32–623	[62,89,91,158]
	*Aspergillus ochraceus*	470	[91]
	*Aspergillus parasiticus*	2500	[64]
	*Candida albicans*	0.32–700	[74,88,89,91,158]
	*Candida glabrata*	350	[88]
	*Candida guillermondii*	0.64–1.25	[89]
	*Candida krusei*	0.64–700	[88,89]
	*Candida parapsilosis*	0.64–170	[88,89]
	*Candida tropicalis*	0.32–700	[88,89]
	*Cladosporium* sp.	0.05–0.3	[173]
	*Cryptococcus neoformans*	0.16–78	[89,158]
	*Epidermophyton floccosum*	0.32–2	[74,89]
	*Fusarium* sp.	0.1–0.5	[173]
	*Malassezia furfur*	1–780	[49,174]
	*Microsporum canis*	0.025–2	[68,74,89]
	*Microsporum gypseum*	0.025–1.25	[68,89]
	*Penicillium* sp.	0.1–0.5	[173]
	*Penicillium chrysogenum*	625	[64]
	*Penicillium corylophilum*	0.625	[165]
	*Penicillium funiculosum*	610	[91]
	*Penicillium ochrochloron*	710	[91]
	*Penicillium verrucosum*	1.1719	[90,91]
	*Trichophyton mentagrophytes*	0.32–1.25	[74,89]
	*Trichophyton rubrum*	0.16–1.25	[49,74,89]
	*Trichophyton tonsurans*	1	[74]
	*Trichosporon beigelii*	0.25	[49]
	*Trichophyton erinacei*	0.5	[68]
	*Trichophyton mentagrophytes*	0.5	[68]
	*Trichophyton terrestre*	0.25	[68]
*Pogostemon cablin* (Blanco) Benth.	*Aspergillus flavus*	>1500	[92]
	*Aspergillus niger*	156	[158]
	*Aspergillus oryzae*	>1500	[92]
	*Candida albicans*	32–625	[158,175]
	*Candida krusei*	64–257	[175]
	*Candida tropicalis*	32–257	[175]
	*Cryptococcus neoformans*	20	[158]
*Pogostemon heyneanus* Benth.	*Candida albicans*	6000	[176]
	*Candida glabrata*	6000	[176]
	*Candida tropicalis*	10,000	[176]
*Premna microphylla* Turcz.	*Aspergillus niger*	>500	[94]
	*Candida albicans*	>500	[94]
	*Fusarium oxysporum*	>500	[94]
*Rosmarinus officinalis* L.	*Aspergillus flavus*	330	[91]
	*Aspergillus ochraceus*	590	[91]
	*Aspergillus niger*	380–10,000	[91,98,158]
	*Botrytis cinerea*	2500	[87]
	*Candida albicans*	30.2–1000	[51,91,96,98,158]
	*Cryptococcus neoformans*	313	[158]
	*Epidermophyton floccosum*	30	[96]
	*Microsporum canis*	2.5–30.2	[68,96]
	*Microsporum gypseum*	2.5	[68]
	*Penicillium expansum*	5000	[87]
	*Penicillium ochrochloron*	470	[91]
	*Penicillium funiculosum*	570	[91]
	*Trichophyton erinacei*	1.5	[68]
	*Trichophyton mentagrophytes*	5–15.3	[68,96]
	*Trichophyton rubrum*	15–256	[51,96]
	*Trichophyton terrestre*	5	[68]
	*Trichophyton tonsurans*	15.2	[96]
*Salvia fruticosa* Miller	*Candida albicans*	512	[51]
	*Fusarium oxysporum* f. sp. *dianthi*	>2000	[99]
	*Fusarium proliferatum*	>2000	[99]
	*Fusarium solani* f. sp. *cucurbitae*	>2000	[99]
	*Malassezia furfur*	>4	[99]
	*Rhizoctonia solani*	>2000	[99]
	*Sclerotinia sclerotiorum*	>2000	[99]
	*Trichophyton rubrum*	2–256	[49,99]
	*Trichosporon beigelii*	4	[49]
*Salvia mirzayanii* Rech. f. and Esfand	*Candida albicans*	0.5–2	[100]
	*Candida krusei*	1	[100]
	*Candida dubliniensis*	0.06–0.5	[100]
	*Candida glabrata*	0.06–1	[100]
	*Candida parapsilosis*	0.25–1	[100]
	*Candida tropicalis*	0.25–2	[100]
	*Trichosporon* sp.	1	[100]
*Salvia officinalis* L.	*Aspergillus flavus*	5–10	[101]
	*Aspergillus fumigatus*	2.5–5	[101]
	*Aspergillus niger*	5–1250	[101,158]
	*Candida albicans*	2.5–2780	[96,101,158,177]
	*Candida guillermondii*	1.25–2.5	[101]
	*Candida krusei*	2.5–5	[101]
	*Candida parapsilosis*	5	[101]
	*Candida tropicalis*	5	[101]
	*Cryptococcus neoformans*	0.64–625	[101,158]
	*Epidermophyton floccosum*	0.64–100	[96,101]
	*Microsporum canis*	1.25–100.2	[96,101]
	*Microsporum gypseum*	1.25–2.5	[101]
	*Trichophyton mentagrophytes*	1.25–60	[96,101]
	*Trichophyton mentagrophytes var. interdigitale*	1.25	[101]
	*Trichophyton rubrum*	0.64–60	[96,101]
	*Trichophyton tonsurans*	60	[96]
	*Trichophyton verrucosum*	1.25–2.5	[101]
*Salvia sclarea* L.	*Aspergillus niger*	1250	[158]
	*Candida albicans*	1250	[158]
	*Cryptococcus neoformans*	313	[158]
	*Fusarium delphinoides*	1800–3600	[85]
	*Fusarium incarnatum-equiseti*	1800–3600	[85]
	*Fusarium napiforme*	1800–3600	[85]
	*Fusarium oxysporum*	1800–3600	[85]
	*Fusarium solani*	3600–7200	[85]
	*Fusarium verticillioides*	1800	[85]
*Satureja hortensis* L.	*Alternaria alternata*	62.5	[103]
	*Aspergillus flavus*	31.25–500	[103,104,117]
	*Aspergillus niger*	471	[117]
	*Aspergillus ochraceus*	423	[117]
	*Aspergillus parasiticus*	373	[117]
	*Aspergillus terreus*	389	[117]
	*Aspergillus variecolor*	125	[103]
	*Candida albicans*	200–400	[103,178]
	*Fusarium culmorum*	125	[103]
	*Fusarium oxysporum*	250	[103]
	*Microsporum canis*	62.5	[103]
	*Moniliania fructicola*	31.25	[103]
	*Penicillium* spp.	125	[103]
	*Rhizoctonia solani*	125	[103]
	*Rhizopus* spp.	250	[103]
	*Sclerotinia minor*	250	[103]
	*Sclerotinia sclerotiorum*	125	[103]
	*Trichophyton mentagrophytes*	62.5	[103]
	*Trichophyton rubrum*	31.25	[103]
*Satureja montana* L.	*Microsporum canis*	0.5	[68]
	*Microsporum gypseum*	2	[68]
	*Trichophyton erinacei*	2	[68]
	*Trichophyton mentagrophytes*	2	[68]
	*Trichophyton terrestre*	3	[68]
*Satureja thymbra* L.	*Aspergillus flavus*	25	[105]
	*Aspergillus fumigatus*	1.25–25	[105,179]
	*Aspergillus niger*	2.5–25	[105,179]
	*Aspergillus ochraceus*	2.5–25	[105,179]
	*Aspergillus versicolor*	1.25	[179]
	*Candida albicans*	25–128	[51,105]
	*Penicillium funiculosum*	2.5–25	[105,179]
	*Penicillium ochrochloron*	1–1.25	[105,179]
	*Trichoderma viride*	1.25–25	[105,179]
	*Trichophyton rubrum*	128	[51]
*Stachys cretica* L.	*Candida albicans*	625	[106]
*Stachys officinalis* (L.) Trevis	*Aspergillus niger*	2500	[107]
	*Candida albicans*	5000	[107]
*Stachys pubescens* Ten.	*Alternaria alternata*	1	[108]
	*Aspergillus flavus*	0–5	[108]
	*Fusarium oxysporum*	1	[108]
*Teucrium sauvagei* Le Houerou	*Aspergillus fumigatus*	>1000	[109]
	*Candida albicans*	>1000	[109]
	*Cryptococcus neoformans*	>1000	[109]
	*Epidermophyton floccosum*	850	[109]
	*Microsporum canis*	800	[109]
	*Microsporum gypseum*	900	[109]
	*Scopulariopsis brevicaulis*	>1000	[109]
	*Scytalidium dimidiatum*	>1000	[109]
	*Trichophyton mentagrophytes* var. *interdigitale*	950	[109]
	*Trichophyton mentagrophytes* var. *mentagrophytes*	900	[109]
	*Trichophyton rubrum*	800	[109]
	*Trichophyton soudanense*	800	[109]
*Teucrium yemense* Deflers.	*Aspergillus niger*	313	[77]
	*Botrytis cinerea*	313	[77]
	*Candida albicans*	1250	[77]
*Thymbra capitata* (L.) Cav. = *Thymus capitatus* (L.) Hoffmanns. and Link = *Coridothymus capitatus* (L.) Rchb.f. Solms	*Aspergillus flavus*	0.32	[111]
	*Aspergillus fumigatus*	0.16–0.32	[111]
	*Aspergillus niger*	0.1–0.16	[111,180]
	*Aspergillus oryzae*	0.2	[180]
	*Candida albicans*	0.16–128	[51,110,111,112]
	*Candida glabrata*	0.32	[111,112]
	*Candida guilliermondii*	0.16–0.32	[111,112]
	*Candida krusei*	0.32	[111]
	*Candida parapsilosis*	0.32	[111,112]
	*Candida tropicalis*	0.32	[111,112]
	*Epidermophyton floccosum*	0.08	[111]
	*Fusarium solani*	0.2	[180]
	*Microsporum canis*	0.08	[111]
	*Microsporum gypseum*	0.08	[111]
	*Penicillium digitatum*	0.5	[180]
	*Trichophyton mentagrophytes*	0.08	[111]
	*Trichophyton rubrum*	0.16–64	[51,111]
*Thymbra spicata* L.	*Aspergillus fumigatus*	0.3	[179]
	*Aspergillus niger*	0.6	[179]
	*Aspergillus versicolor*	0.3	[179]
	*Aspergillus ochraceus*	0.6	[179]
	*Candida albicans*	1.12–3750	[51,113,114]
	*Candida krusei*	1.12	[114]
	*Candida parapsilosis*	0.6–1.12	[114]
	*Penicillium funiculosum*	0.3	[179]
	*Penicillium ochrochloron*	0.3	[179]
	*Trichoderma viride*	0.3	[179]
	*Trichophyton rubrum*	64	[51]
*Thymus bovei* Benth.	*Candida albicans*	250	[115]
*Thymus daenensis* Celak.	*Alternaria alternata*	>8	[108]
	*Aspergillus flavus*	1	[108]
	*Fusarium oxysporum*	4	[108]
*Thymus kotschyanus* Boiss. and Hohen.	*Alternaria alternata*	1	[108]
	*Aspergillus flavus*	0.5	[108]
	*Fusarium oxysporum*	0–5	[108]
*Thymus mastichina* (L.) L.	*Candida albicans*	1.25–2.5	[116]
	*Candida glabrata*	1.25–1.5	[116]
	*Candida guilliermondii*	1.25	[116]
	*Candida krusei*	1.25–2.5	[116]
	*Candida parapsilosis*	2.5–5	[116]
	*Candida tropicalis*	2.5–10	[116]
*Thymus migricus* Klokov et Des.-Shost.	*Aspergillus flavus*	452	[117]
	*Aspergillus niger*	460	[117]
	*Aspergillus ochraceus*	430	[117]
	*Aspergillus parasiticus*	581	[117]
	*Aspergillus terreus*	447	[117]
*Thymus pulegioides* L.	*Aspergillus flavus*	0.32	[119]
	*Aspergillus fumigatus*	0.16	[119]
	*Aspergillus niger*	0.32	[119]
	*Candida albicans*	0.32–0.64	[119]
	*Candida glabrata*	0.32–0.64	[119]
	*Candida guilliermondii*	0.32	[119]
	*Candida krusei*	0.32–0.64	[119]
	*Candida parapsilosis*	0.64	[119]
	*Candida tropicalis*	0.32–0.64	[119]
	*Epidermophyton floccosum*	0.16	[119]
	*Microsporum canis*	0.16	[119]
	*Microsporum gypseum*	0.16	[119]
	*Trichophyton mentagrophytes*	0.16	[119]
	*Trichophyton rubrum*	0.32	[119]
*Thymus schimperi* Ronninger	*Aspergillus minutus*	0.512–2	[120]
	*Aspergillus niger*	0.16	[181]
	*Aspergillus tubingensis*	1–4	[120]
	*Beauveria bassiana*	0.128–1	[120]
	*Candida albicans*	0.16	[181]
	*Microsporum* spp.	0.08	[181]
	*Microsporum gypseum*	0.128–1	[120]
	*Penicillium chrysogenum*	0.512–2	[120]
	*Rhodotorula* spp.	0.08	[181]
	*Tricophyton* spp.	0.08–0.31	[181]
	*Verticillium* sp.	0.512–2	[120]
*Thymus serpyllum* L.	*Aspergillus carbonarius*	1.25	[182]
	*Aspergillus ochraceus*	0.625	[182]
	*Aspergillus niger*	2.5	[182]
	*Microsporum canis*	0.025	[68]
	*Microsporum gypseum*	0.25	[68]
	*Trichophyton erinacei*	0.1	[68]
	*Trichophyton mentagrophytes*	0.2	[68]
	*Trichophyton terrestre*	0.1	[68]
*Thymus striatus* Vahl.	*Alternaria alternata*	1	[121]
	*Aspergillus flavus*	1.5	[121]
	*Aspergillus niger*	1	[121]
	*Aspergillus ochraceus*	1	[121]
	*Aspergillus terreus*	1	[121]
	*Aspergillus versicolor*	1	[121]
	*Cladosporium cladosporioides*	0.5	[121]
	*Epidermophyton floccosum*	1	[121]
	*Microsporum canis*	1.5	[121]
	*Penicillium funiculosum*	2	[121]
	*Penicillium ochrochloron*	2	[121]
	*Phomopsis helianthi*	0.5	[121]
	*Trichoderma viride*	2	[121]
	*Trichophyton mentagrophytes*	1	[121]
*Thymus vulgaris* L.	*Absidia* spp.	7 ± 4	[122]
	*Alternaria* spp.	9.4 ± 4.5	[122]
	*Alternaria alternata*	4.7–500	[122,183]
	*Aspergillus* spp.	3.2	[122]
	*Aspergillus flavus*	9.35–1500	[64,104,122,125,184]
	*Aspergillus fumigatus*	144–1000	[124,184]
	*Aspergillus niger*	9.35–1250	[64,122,158,184]
	*Aspergillus ochraceus*	2.5–750	[164,184]
	*Aspergillus parasiticus*	1250	[64]
	*Aspergillus sulphureus*	10.88 ± 3.1	[122]
	*Aspergillus versicolor*	9.6 ± 9.25	[122]
	*Botrytis cinerea*	312	[87]
	*Candida albicans*	0.16–313	[73,74,116,158]
	*Candida glabrata*	0.16–0.32	[116]
	*Candida krusei*	0.08–0.16	[116]
	*Candida guillermondii*	0.16	[116]
	*Candida parapsilosis*	0.16–0.32	[116]
	*Candida tropicalis*	0.16–0.32	[116]
	*Chaetomium globosum*	1.6	[122]
	*Cladosporium* spp.	12.8	[122]
	*Cladosporium sphaerospermum*	19.6	[122]
	*Cryptococcus neoformans*	78	[158]
	*Epidermophyton floccosum*	4	[74]
	*Fusarium* spp.	62.5	[185]
	*Fusarium delphinoides*	900–1800	[85]
	*Fusarium incarnatum-equiseti*	450–3600	[85]
	*Fusarium napiforme*	900	[85]
	*Fusarium oxysporum*	5–900	[85,126]
	*Fusarium solani*	1800–3600	[85]
	*Fusarium verticillioides*	900	[85]
	*Malassezia furfur*	920	[174]
	*Microsporum canis*	2.2	[74]
	*Mortierella* spp.	250	[185]
	*Mucor* spp.	50.2 ± 8.4	[122]
	*Penicilium* spp.	18.95–500	[122,185]
	*Penicilium brevicompactum*	19.6	[122]
	*Penicillium chrysogenum*	312.5–1750	[64,184]
	*Penicilium chrysogenum*	19.6	[122]
	*Penicillium citrinum*	1250	[184]
	*Penicillium expansum*	625	[87]
	*Penicillium griseofulvum*	19.6	[122]
	*Rhizopus* spp.	12.6	[122]
	*Rhodotorula glutinis*	72	[73]
	*Rhizopus oryzae*	256–512	[123]
	*Saccharomyces cerevisiae*	72	[73]
	*Schizosaccharomyces pombe*	36	[73]
	*Stachybotrys chartarum*	6.2	[122]
	*Trichoderma* spp.	16.8	[122]
	*Trichophyton mentagrophytes*	2.2	[74]
	*Trichophyton rubrum*	2–72	[74,124]
	*Trichophyton tonsurans*	2.2	[74]
	*Ulocladium* spp.	5.45 ± 1.5	[122]
	*Yarrowia lypolytica*	36	[73]
*Thymus zygis* L.	*Candida albicans*	0.16–0.32	[116]
	*Candida glabrata*	0.32	[116]
	*Candida krusei*	0.16–0.32	[116]
	*Candida guillermondii*	0.16	[116]
	*Candida parapsilosis*	0.32	[116]
	*Candida tropicalis*	0.16–0.32	[116]
	*Penicillium corylophilum*	0.3125–0.625	[165]
*Vitex agnus-castus* L.	*Candida albicans*	0.53–512	[51,129]
	*Candida dubliniensis*	0.27	[129]
	*Candida famata*	2.13	[129]
	*Candida glabrata*	0.27	[129]
	*Candida krusei*	0.27	[129]
	*Candida lusitaniae*	2.13	[129]
	*Candida parapsilosis*	1.06	[129]
	*Candida tropicalis*	0.13	[129]
	*Epidermophyton floccosum*	0.64–2.5	[128]
	*Microsporum canis*	0.64–5	[128]
	*Microsporum gypseum*	1.25–10	[128]
	*Trichophyton mentagrophytes*	1.25–10	[128]
	*Trichophyton rubrum*	0.64–512	[51,128]
*Zataria multiflora* Boiss.	*Aspergillus flavus*	358	[117]
	*Aspergillus niger*	358	[117]
	*Aspergillus ochraceus*	341	[117]
	*Aspergillus parasiticus*	367	[117]
	*Aspergillus terreus*	447	[117]
	*Microsporum canis*	0.125–0.25	[130]
	*Microsporum gypseum*	0.03–0.06	[130]
	*Trichophyton mentagrophytes*	0.03	[130]
	*Trichophyton rubrum*	0.03–0.06	[130]
	*Trichophyton schoenleinii*	0.125–0.6	[130]
*Ziziphora clinopodioides* Lam.	*Aspergillus flavus*	48.82	[184,186]
	*Aspergillus fumigatus*	1750	[184]
	*Aspergillus niger*	3000	[184]
	*Aspergillus ochraceus*	1500	[184]
	*Aspergillus parasiticus*	48.82	[186]
	*Penicillium chrysogenum*	3000	[184]
	*Penicillium citrinum*	1750	[184]
*Ziziphora tenuior* L.	*Aspergillus flavus*	1.25	[133]
	*Aspergillus fumigatus*	0.64	[133]
	*Aspergillus niger*	0.64	[133]
	*Candida albicans*	1.25	[133]
	*Candida guillermondii*	1.25	[133]
	*Candida krusei*	1.25	[133]
	*Candida parapsilosis*	1.25	[133]
	*Candida tropicalis*	1.25	[133]
	*Cryptococcus neoformans*	0.16	[133]
	*Epidermophyton floccosum*	0.64	[133]
	*Microsporum canis*	0.64–1.25	[133]
	*Microsporum gypseum*	1.25	[133]
	*Trichophyton mentagrophytes*	1.25	[133]
	*Trichophyton mentagrophytes* var. *interdigitale*	1.254	[133]
	*Trichophyton rubrum*	0.64	[133]
	*Trichophyton verrucosum*	0.64	[133]
